# First report of *T. spiralis* in a wolf in Italy: An increasing health concern?

**DOI:** 10.1016/j.fawpar.2024.e00253

**Published:** 2024-12-26

**Authors:** G. Marucci, C. Raso, E. Borgogni, F. Celani, I. Tartarelli, S. Cherchi, A. Di Giambattista, P. Calderini, A. Casulli

**Affiliations:** a Unit of Foodborne and Neglected Parasitic Diseases, Department of Infectious Diseases, Istituto Superiore di Sanità, Viale Regina Elena 299, 00161 Rome, Italy; b European Union Reference Laboratory for Parasites (EURL-P), Istituto Superiore di Sanità, Viale Regina Elena 299, 00161 Rome, Italy; c Istituto Zooprofilattico Sperimentale del Lazio e della Toscana, Via Tancia, 21, 02100 Rieti, Italy; d Azienda Sanitaria Locale di Rieti, Via del Terminillo,42, 02100 Rieti, Italy

**Keywords:** *Trichinella spiralis*, Wolf (*Canis lupus*), Trichinellosis, Italy

## Abstract

*Trichinella spiralis* is a zoonotic nematode parasite of worldwide distribution. It is present in Europe with important foci, particularly in Eastern countries and Spain. This species is generally associated with a domestic cycle that involves primarily pigs. It is best adapted for pigs but can also infect a wide range of other domestic, synanthropic, and wild mammals including carnivores, omnivores and scavengers. Before 2016, when *T. spiralis* larvae were detected in a red fox (*Vulpes vulpes*) in the Piacenza province (Emilia Romagna region, Northern Italy), this parasite had only been reported in Italy occasionally, being found in horses or pork products imported from Eastern Europe. We describe here the first isolation of *T. spiralis* in a wolf (*Canis lupus*) in the Lazio region, Central Italy. In the wolf specimen *T. spiralis* was identified in coinfection with *Trichinella britovi*, a species endemic in Italian wildlife. Among the *Trichinella* species, *T. spiralis* is the most frequently associated with human disease in Europe and is known to cause more severe symptoms than *T. britovi*. In light of wolf population expansion, the detection of *T. spiralis* in Central Italy implies new scenarios for the risk of human trichinellosis because of the high risk this species represents for domestic and wild pigs. Active monitoring of wildlife living in these areas is necessary to define the actual distribution of  this species and to detect its possible presence in other areas of the Italian peninsula.

## Highlights

• *Trichinella spiralis* has been identified for the first time in a wolf in Italy.
• This species represents a high risk for domestic and wild swine.
• The presence of *Trichinella spiralis* in Italian wild fauna is an increasing health concern.

## Graphical abstract

**Figure:**
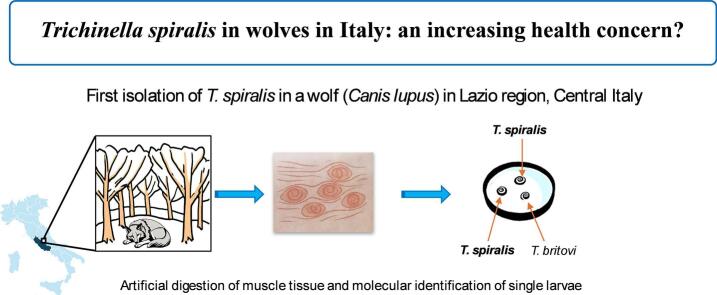

## 1 Introduction

Nematodes of the genus *Trichinella* are food-borne zoonotic agents causing trichinellosis in humans and having a global distribution in domestic and wild animals (Pozio and Darwin, 2006; Pozio et al., 2009). The main reservoir hosts of these pathogens are carnivorous and omnivorous mammals; however, some species are also able to infect birds (*Trichinella pseudospiralis*) and reptiles (*Trichinella papuae* and *Trichinella zimbabwensis*). *Trichinella* spp. are widespread on all continents, except Antarctica (Pozio, 2019). In Europe, four species with different biological and ecological characteristics, namely *Trichinella nativa*, *T. spiralis*, *T. britovi* and *T. pseudospiralis*, are known to circulate in wildlife and some of them also circulate in sylvatic and domestic pigs (Bilska-Zając et al., 2020; EFSA/ECDC, 2023; Marucci et al., 2022).

*Trichinella britovi* is the predominant species present in the wild fauna of Italy, followed by *T. pseudospiralis,* which has been so far reported in tawny owl (*Strix aluco*), little owl (*Athene noctua*), red kite (*Milvus milvus*), western marsh harrier (*Circus aeruginosus*), wild boar, red foxes (*Vulpes vulpes)* and Italian wolf (*Canis lupus italicus*), and then by *T. spiralis,* for which sporadic infections has been reported in red foxes (Garbarino et al., 2017; Marucci et al., 2021; Merialdi et al., 2011; Ricchiuti et al., 2021; Rugna et al., 2022). *Trichinella spiralis* constitutes a major health concern for humans, as it reaches higher burden in pigs than do other parasites of its genus (Pozio et al., 2009); further, *T. spiralis* is responsible for most of human outbreaks of trichinellosis in Europe (EFSA/ECDC, 2023).

The wolf (*Canis lupus*) is the largest member of Canidae native to Eurasia and North America (Carbyn et al., 1995). According to a report prepared by the Large Carnivore Initiative for Europe in 2022, there are currently around 19,000 wolves distributed across the 27 EU Member States, and 21,500 across Europe as a whole (Boitani et al., 2022). This carnivore, at the top of the food chain, is an excellent predator but also a scavenger (Zlatanova et al., 2014) and may thus play an important role in maintaining *Trichinella* spp. in the wild. Moreover, the ability of wolves to range over a large territory, often covering long distances (Morales-González et al., 2022), could allow *Trichinella* spp. to colonise new areas. According with EFSA/ECDCEuropean Union One Health 2022 Zoonoses Report, among wolves tested in Italy in 2022, 10.1 % were positive for *Trichinella* spp. (EFSA/ECDC, 2023).

Until the mid-19th century, the wolf was widespread throughout the Italian mainland and in Sicily, particularly in the mountainous areas of the Apennines and the Alps. In the following decades, however, it underwent a dramatic numerical reduction and range contraction, due to anthropic activities. This reduction in prevalence and range brought the Italian wolf population, considered as a pest animal, on the verge of extinction in the early seventies. Since 1971 with the ministerial decree “Natali”(Decreto ministeriale 1° luglio 1971 del Ministero dell'Agricoltura e delle Foreste, 1971), legal protection against unregulated culling, abandonment of marginal rural areas, the increased availability of undisturbed areas, and a gradual increase of wild prey (Chapron et al., 2014; Cimatti et al., 2021) wolves returned to colonise part of their former territory. Currently, this species is known to inhabit the whole Apennine chain, the western Alps and the central alpine sector (Galaverni et al., 2016; ISPRA, 2022).

The recolonisation of Italy by wolves led to an increased interest in defining the role of this species as a reservoir, carrier and sentinel of infectious agents. Being an apex predator with a long-life span, the wolf can be a sentinel of neglected infectious agents that are circulating in the wild. Moreover, having a home range of 200–400 km^2^ (Boitani, 1992) and a maximum dispersal ability up to 850 km (Dondina et al., 2020), the wolf could also play a role in the transmission and long-distance spread of pathogens (Marucco et al., 2022). This case report aims to document the first detection of *T. spiralis* in a wolf in Italy in the light of its public health relevance.

## 2 Materials and methods

**Fig. 1:**
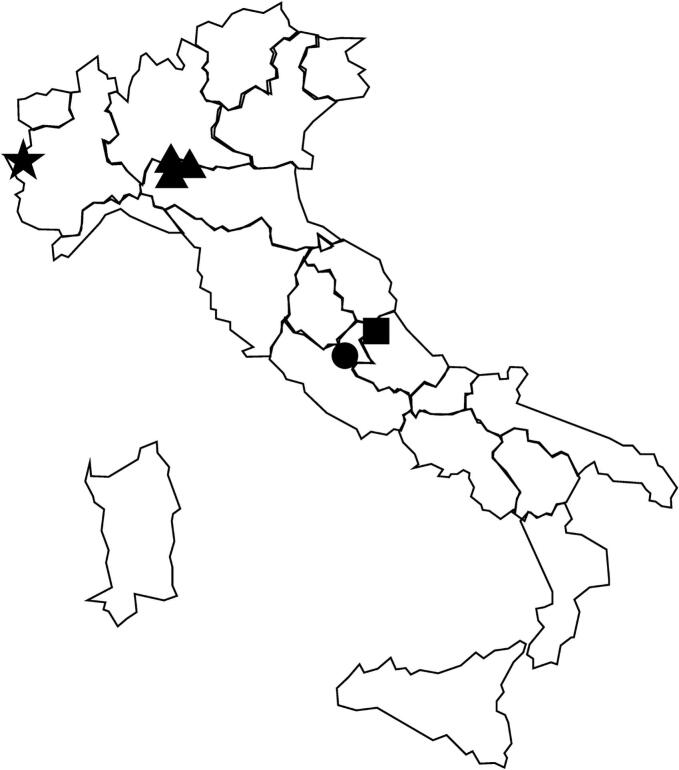
Map of *Trichinella spiralis* infected wild animals sampled in Italy. ★, red fox collected in Bardonecchia municipality in 1991; ▲, red foxes collected in Piacenza municipality in 2016, 2017 and 2018; ■, wolf collected in Campotosto municipality in 2022; ●, wolf collected in Cittaducale municipality in 2024. (For interpretation of the references to colour in this figure legend, the reader is referred to the web version of this article.)

In July 2024, a 35 kg adult male wolf (*Canis lupus*) was found dead in the Cittaducale municipality (Rieti province, Lazio region, Italy) (Fig. 1). The carcass was transferred to the Diagnostic Laboratory of the Istituto Zooprofilattico Sperimentale del Lazio e della Toscana for routine necroscopy. The wolf carcass was tested for the presence of chemical and infective agents, including the presence of *Trichinella* spp. muscle larvae. About 30 g of muscle from the tibialis cranial muscle were tested by artificial digestion according to the magnetic stirrer method for pooled sample digestion, the gold standard for *Trichinella* spp. detection (ISO 18743:2015/Amd 1:2023). After artificial digestion, larvae were collected, counted, stored in 96 % ethanol, and sent to the European Union Reference Laboratory for Parasites (EURL-P; https://www.iss.it/en/eurlp-chi-siamo) (Rome, Italy) for species identification by multiplex PCR (Pozio and Zarlenga, 2019). Briefly, DNA was purified from single larvae using a DNA IQ System kit (Promega, Madison, WI, USA) and a Tissue and Hair Extraction kit (Promega, USA). Five primer sets, targeting specific regions (expansion segment V, ITS1 and ITS2) of the ribosomal DNA repeats, were used in multiplex PCR to obtain a species-specific electrophoretic DNA banding pattern (Zarlenga et al., 1999; Pozio and La Rosa, 2010).

## 3 Results and discussion

Artificial digestion of the wolf muscle resulted in the recovery of 30 *Trichinella* muscle larvae (1 larva per gram of tissue). The first identification made by multiplex PCR on six single larvae showed the presence of a mixed infection involving *T. spiralis* and *T. britovi.* To investigate the proportion of *T. spiralis* and *T. britovi* larvae in the isolate, the remaining 24 larvae were tested. In total, 22 larvae (73 %) were identified as *T. spiralis*, five larvae (17 %) were identified as *T. britovi*, and three larvae (10 %) tested negative by multiplex PCR.

*Trichinella spiralis* has a higher larval burden and a longer survival time in domestic and wild pigs as compared with other *Trichinella* species (Pozio and Darwin, 2006). Because of the higher infectivity of *T. spiralis* for pigs, it is the main etiological agent of trichinellosis in humans worldwide (Murrell and Pozio, 2011).

*Trichinella spiralis* is believed to have originated in Eastern Asia and to have been introduced in Europe by translocation of domestic pigs from the Far East; then, thanks to European colonization, it was spread to other continents (Pozio and Zarlenga, 2021). Currently, the distribution of *T. spiralis* in pigs is linked to backyard and free-ranging pig husbandry, practices generally corresponding to less developed regions. Nevertheless, this species also circulates in wild animals, both omnivores and carnivores, which can play important roles as reservoirs. In fact, out of more than 3500 *T. spiralis* isolates identified so far at the International Trichinella Reference Center (ITRC), 35 % originated from domestic pig, 55 % originated from wild boars and 10 % from other carnivores (Marucci et al., 2022; *Trichinella* database of the ITRC).

*Trichinella britovi* is the predominant species infecting wild carnivores across the entire European continent, Western Asia and North-Western Africa, excluding some islands (e.g., Great Britain and Ireland) (Pozio, 2019). This species is most commonly reported in wild boar and red fox, but has also been reported in canids (e.g. wolf; jackal, *Canis aureus*), viverrids (e.g., European genet, *Genetta genetta*; African palm civet, *Nandina binotata*), mustelids (e.g., stone marten, *Martes foina*; badger, *Meles meles*), rodents (beaver, *Castor fiber*; rats, *Rattus* spp.), and Ursidae (brown bear, *Ursus arctos*) (Pozio, 2005; Pozio et al., 2009; Marucci et al., 2022).

Before its identification, in the present work, in a wolf in the Rieti province, *T. spiralis* was reported in wildlife only few times in Italy. In 1991, it was reported in a red fox (*Vulpes vulpes*) hunted in the Bardonecchia municipality (Turin province, Piedmont region**;** close to the French border), and in 2016, 2017 and 2018 in single red fox specimens collected in the Travo municipality (Piacenza Province, Emilia Romagna region) (Garbarino et al., 2017). The discovery of the fox infected with *T. spiralis* in Bardonecchia was explained as an accidental occurrence, due to the proximity to French border. The three foxes infected by *T. spiralis* collected in the Travo municipality, in three successive years, all originated from the same locality, a sparsely populated area in the Trebbia River valley. No other wild animals tested in the area were found to be positive for *Trichinella* spp.; further, no open-air landfills or waste dumping sites were detected, and the local population had no official trade with Eastern Europe. Consequently, the authors hypothesized that dogs played a role in this transmission route, as some dogs in the area were imported or used for hunting in the Eastern European countries, where *T. spiralis* is endemic. According to this hypothesis, foxes contracted the infection by feeding on an infected dog carcass drifting after the flood of the Trebbia River in September 2015. (Garbarino et al., 2017; Garbarino, 2019).

Occasional introduction of *T. spiralis* in infected meat or animals has occurred in Italy in the past. In fact, three *T. spiralis* infected horses, imported from eastern Europe and slaughtered in Italy, were the source of outbreaks of trichinellosis in the Apulia region (Southern Italy) in 1990 and in 2000, and in Piacenza (Emilia-Romagna region, Northern Italy) in 1998 (Pozio, 2015). Moreover, in 2000 and in 2002, two human cases were reported due consumption of *T. spiralis* infected pork sausages imported in personal baggage into Italy from Serbia and Romania (Pozio and Marucci, 2003).

The recent identification of *T. spiralis* in a wolf in central Italy could have a significant impact on public health risk as the wolf has not previously been considered a reservoir host for this species. At the national level, the estimated wolf population is about 3300 individuals (ISPRA, 2022). In the last 10 years (2013−2023), 239 wolf specimens (14 %) from a total of 1724 tested animals were positive for *Trichinella* sp. The identification at the species level showed that 112 wolves (47 %) were infected by *T. britovi*, one (0.4 %) had a *T. britovi*/*T. pseudospiralis* mixed infection, while the remaining 126 *Trichinella* isolates (53 %) could not be identified. In the same period, in other European countries, a total of 210 wolf specimens (25 %) have been found positive to *Trichinella* sp. over a total of 825 tested animals. Of the 210 positive animals, 22 (10 %) were infected with *T. nativa*, 22 (10 %) with *T. britovi*, while for 166 specimens (79 %) the *Trichinella* species has not been identified (EFSA/ECDC, 2015a, EFSA/ECDC, 2015b, EFSA/ECDC, 2016, EFSA/ECDC, 2017, EFSA/ECDC, 2018, EFSA/ECDC, 2019, EFSA/ECDC 2021a, EFSA/ECDC, 2021b, EFSA/ECDC, 2022, EFSA/ECDC, 2024, EFSA/ECDC, 2023).

According to data available both in the literature and in the ITRC database, *T. spiralis* has been detected in a wolf only ten times (Table 1), a very few number of reports compared to what has been observed for *T. britovi* and *T. nativa*, which have been isolated in 285 and 147 wolf specimens, respectively (ITRC database).

**Table 1:** *Trichinella spiralis* isolates detected in wolves in Europe. Data obtained from literature and from ITRC database.

Year	Country	Region/Province	Reference
1976	Spain	Galicia	International Trichinella Reference Center database
2009	Croatia	Dalmatia	Beck et al., 2009
2009	Croatia	Gorski Kotar	Beck et al., 2009
2011	Serbia	Smederevo	International Trichinella Reference Center database
2011	Germany	Brandenburg	International Trichinella Reference Center database
2011	Finland	Päijät-Häme	International Trichinella Reference Center database
2013	Finland	South Ostrobothnia	International Trichinella Reference Center database
2014	Russia	Kirov Oblast	International Trichinella Reference Center database
2022	Sweden	Varmland	International Trichinella Reference Center database
2024	Italy	Latium	Present work

In December 2022, a few *Trichinella* larvae were isolated from muscle tissue of a wolf collected in the Campotosto municipality (L'Aquila province, Abruzzo region, Italy). These larvae were tested by multiplex PCR and a weak band attributable to *T. spiralis* was observed in some of them. Unfortunately, it was not possible to repeat the test and have confirmation of the observed result because of lack of biological material; thus, the *Trichinella* species remained unidentified. In light of what we observed in the wolf specimen from Cittaducale, and taking into consideration that the two localities (Cittaducale and Campostosto) are only 40 km apart, we cannot exclude that the first report of *T. spiralis* infection in wolf in Central Italy can be backdated to 2022.

The above-mentioned findings may suggest the presence of a *T. spiralis* focus in Central Italy. We can speculate that *T. spiralis* has recently started to settle in Italian territory, moving from North of Italy, where this species was identified for the first time in 2016, to the Central regions of the Italian peninsula. It is intriguing that no other susceptible wild animal has been found infected with *T. spiralis* in the same area, so far. However, considering the high amount (53 %) of unidentified *Trichinella* isolates collected from wolves in Italy, it is possible that some *T. spiralis* infections passed unnoticed, especially if this species has a very low prevalence.

In addition, given the occasional scavenger feeding habit of wolves at garbage dumps (Zlatanova et al., 2014), a less probable alternative route of introduction through improper disposal of imported infected meat, cannot be totally excluded.

## 4 Conclusions

We report the presence of *T. spiralis* infection in a wolf from Central Italy. This is the first time that *T. spiralis* was reported in a wolf specimen in Italy, confirming the presence of this species in the Italian peninsula. Among the *Trichinella* species, *T. spiralis* is most frequently associated with human disease in Europe and is known to cause more severe symptoms than *T. britovi*, which is endemic in Italian wildlife. The detection of *T. spiralis* coupling with expanding wolf population opens new scenarios for the epidemiology of trichinellosis in Italy and has significant implications for public health, underlining the importance of maintaining an active wildlife surveillance on the territory for early monitoring of emerging infectious agents.

## CRediT authorship contribution statement

**G. Marucci:** Writing – original draft, Methodology, Investigation, Conceptualization. **C. Raso:** Writing – original draft, Methodology, Investigation, Conceptualization. **E. Borgogni:** Writing – original draft, Methodology, Investigation, Conceptualization. **F. Celani:** Writing – original draft, Methodology, Investigation, Conceptualization. **I. Tartarelli:** Writing – original draft, Methodology, Investigation, Conceptualization. **S. Cherchi:** Writing – original draft, Methodology, Investigation, Conceptualization. **A. Di Giambattista:** Writing – original draft, Methodology, Investigation, Conceptualization. **P. Calderini:** Writing – original draft, Methodology, Investigation, Conceptualization. **A. Casulli:** Writing – original draft, Methodology, Investigation, Conceptualization.

## Declaration of competing interest

The authors declare that they have no known competing financial interests or personal relationships that could have appeared to influence the work reported in this paper.
